# Biliary tract infection, abdominal abscess, and postoperative bleeding caused by highly virulent *Klebsiella pneumoniae*: a case report

**DOI:** 10.1093/jscr/rjag380

**Published:** 2026-06-09

**Authors:** Weiyi Sun, Nan Yang, Yanyan Wang, Nan Zhang

**Affiliations:** Department of General Surgery, The First Affiliated Hospital of Henan University of Chinese Medicine, No. 19 Renmin Road, Jinshui District, Zhengzhou, Henan Province, 450000, China; The First Clinical Medical College of Henan University of Chinese Medicine, No. 156 Jinshui East Road, Zhengdong New District, Zhengzhou, Henan Province, 450046, China; The First Clinical Medical College of Henan University of Chinese Medicine, No. 156 Jinshui East Road, Zhengdong New District, Zhengzhou, Henan Province, 450046, China; The Second Affiliated Hospital of Henan University of Chinese Medicine, No. 6 Dongfeng Road, Jinshui District, Zhengzhou, Henan Province, 450002, China; Department of General Surgery, The First Affiliated Hospital of Henan University of Chinese Medicine, No. 19 Renmin Road, Jinshui District, Zhengzhou, Henan Province, 450000, China

**Keywords:** hypervirulent *Klebsiella pneumoniae*biliary tract infection, abdominal abscess, ERCP, laparoscopic cholecystectomy

## Abstract

Hypervirulent *Klebsiella pneumoniae* (hvKp) can cause invasive biliary infection with rapid progression and frequent complications. We report a 70-year-old man with type 2 diabetes and chronic renal insufficiency presenting with abdominal pain. Imaging revealed choledocholithiasis, cholecystitis, and a large intra-abdominal abscess. He underwent ERCP with stone extraction and biliary drainage, followed by laparoscopic cholecystectomy and abscess drainage. Pus culture confirmed *K. pneumoniae* infection. Postoperative stress ulcer bleeding and coagulation dysfunction occurred, but the patient recovered well. This case highlights the importance of early imaging, timely source control, and multidisciplinary management in high-risk patients.

## Introduction

The highly virulent strain of *Klebsiella pneumoniae* (hvKP) has strong invasiveness and dissemination capabilities and has become an important public health threat in recent years [1]. It can cause invasive infections such as liver abscesses, endophthalmitis, and sepsis, among which the liver and gallbladder system is most commonly affected, and diabetic patients are a high-risk group [2, 3]. Biliary tract infection caused by hvKP progresses rapidly and is prone to sepsis, abscess formation and distant dissemination, making clinical diagnosis and treatment difficult. This article reports a case of suspected hvKP caused biliary infection with abdominal abscess and gastrointestinal bleeding after surgery to improve clinical understanding of the disease.

## Case report

**Figure 1:**
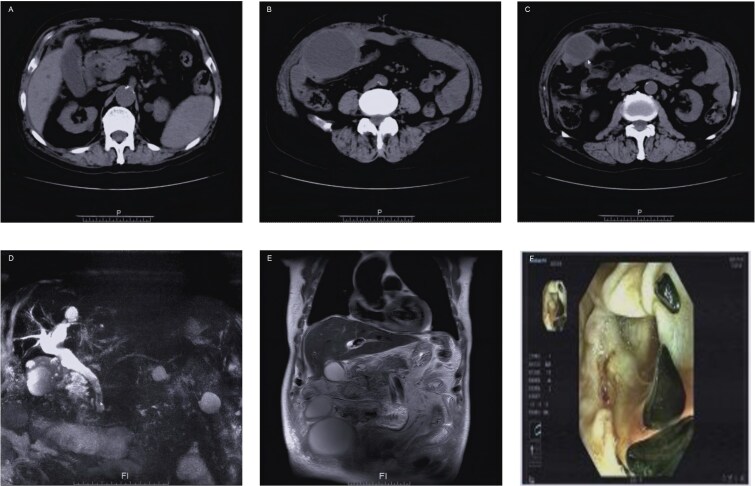
(A, B, C) Plain abdominal CT scan; (D, E) pancreatic bile duct hydrography MRCP; (F) endoscopic retrograde cholangiopancreatography.

**Figure 2:**
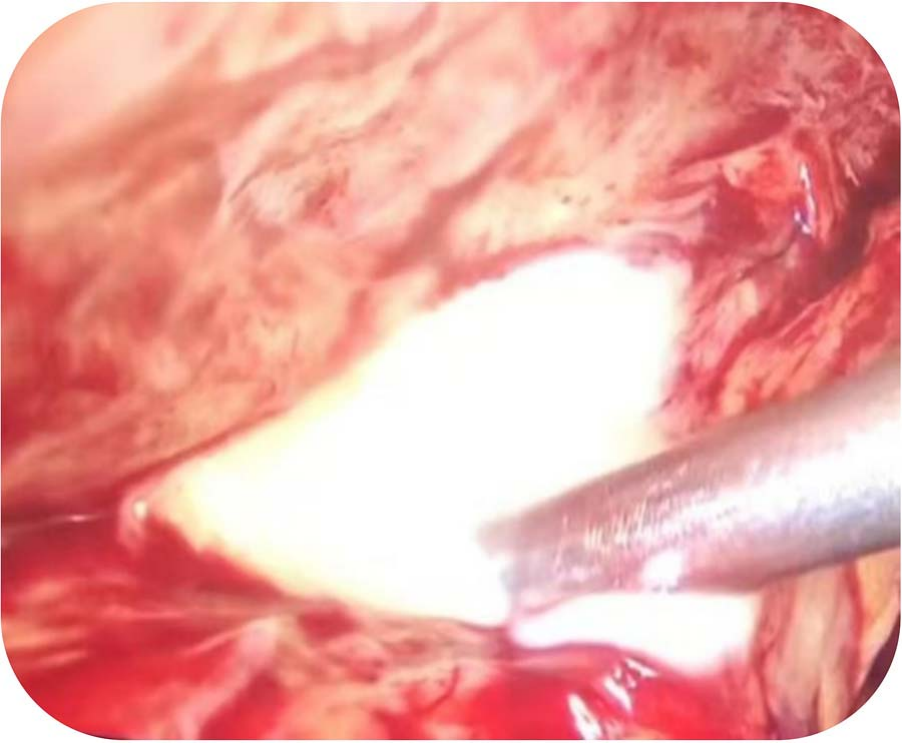
Intraoperative abdominal abscess pus.

The patient, male, 70 years old, was transferred from the Department of Nephrology to our department on 17 October 2025 due to ‘discovery of elevated creatinine for 4 years, accompanied by abdominal pain for 1 day.’ He had a history of IgA nephropathy, chronic renal failure, and type 2 diabetes. He used insulin, and later changed to oral medication by himself. His blood sugar was not well controlled, and he had hypertension, lacunar cerebral infarction, fatty liver, prostate hyperplasia, and renal anemia. Special examination: abdominal distension, tenderness in the right lower abdomen, palpable mass, no rebound pain, bowel sounds 3–4 times/min. Blood routine + inflammation indicators showed: white blood cells 12.41 × 109/L, neutrophil percentage 75.3%, hemoglobin 83 g/L, C-reactive protein 361.38 mg/L, procalcitonin 2.58 ng/mL; biochemistry: creatinine 460.3 μmol/L, urea 38.82 mmol/L, albumin 29.1 g/L. Abdominal computed tomography (CT) demonstrated gallstones with cholecystitis, pericholecystic fat stranding without evidence of gallbladder perforation, dilatation of intrahepatic and extrahepatic bile ducts, a large cystic mass measuring approximately 9.0 × 7.7 × 17 cm in the right abdominal cavity consistent with abscess, and pelvic free fluid (Fig. 1A, B, and  C). MRCP revealed dilatation of intrahepatic and extrahepatic bile ducts, a filling defect in the distal common bile duct consistent with choledocholithiasis, gallbladder stones with cholecystitis, and an abnormal signal collection in the right abdominal cavity consistent with abscess (Fig. 1D and E). The working diagnoses, in order of clinical priority, were choledocholithiasis, acute cholecystitis with gallbladder stones, intra-abdominal abscess, type 2 diabetes mellitus with diabetic nephropathy (stage V) and peripheral vascular disease, chronic renal failure with renal anemia, hypertension (grade 1, very high risk), and superficial venous thrombosis of both lower limbs. On the second day after admission, the patient underwent endoscopic retrograde cholangiopancreatography (ERCP) with endoscopic sphincterotomy (EST), balloon dilation of the bile duct, stone extraction, and placement of an endoscopic nasobiliary drainage (ENBD) tube under combined endoscopic and fluoroscopic guidance (Fig. 1F). On the third day after admission, a gastric tube and catheter were indwelled, and ‘laparoscopic cholecystectomy + incision and drainage of abdominal abscess’ was performed. No gallbladder perforation was found during the operation. The abdominal abscess wrapped a large amount of milky white pus about 200 ml (the same color and viscosity as the pus in the gallbladder), and the pus was taken and sent for culture (Fig. 2). *Klebsiella pneumoniae* was cultured in pus, and drug sensitivity tests showed that sensitive drugs ceftazidime/avibactam (CZA), tigecycline (TGC), compound trimoxazole (TSU), chloramphenicol (CMP), polymyxin B(PB), minocycline (MNO). On the first day after surgery, the patient’s hemoglobin and platelets decreased progressively, with hemoglobin reaching as low as 63 g/L and platelets reaching as low as 64 × 10^9^/L; D-dimer increased significantly, with the highest as 22.37 μg/mL; prothrombin time was prolonged, and about 150 ml of light red fluid was drained from the gastric tube. Postoperative stress ulcer bleeding combined with coagulation disorder was suspected on the basis of blood-tinged nasogastric tube drainage; upper endoscopy was not performed at that time given the patient’s clinical instability, and the diagnosis therefore remains presumptive. After symptomatic and supportive treatments such as infusion of suspended red blood cells, plasma, and application of hemostatic drugs, hemoglobin gradually rose to 81 g/L. The patient did not have fever during his hospitalization. Integrated traditional Chinese and Western medicine management was employed: oral administration of a modified Da Chai Hu Tang decoction was prescribed to augment Qi and strengthen the middle energizer, and CRP dropped from 67.5 mg/L to 17.4 mg/L; renal function recovered, and creatinine dropped from 338.2 μmol/L to 248.1 μmol/L. He was discharged after his condition stabilized.

## Discussion

*Klebsiella pneumoniae* is a major pathogen causing severe, life-threatening infections and is classified into classical (cKp) and hypervirulent (hvKp) pathotypes. hvKp is increasingly prevalent in the Asia-Pacific region, whereas cKp predominates in Western countries. hvKp often causes multifocal or metastatic infections and may lead to life-threatening manifestations, particularly in immunocompromised hosts [4–8]. Compared with cKp and other Enterobacteriaceae, hvKp has a greater propensity to cause central nervous system infection and endophthalmitis, highlighting the need for rapid identification and targeted treatment to optimize prognosis.

This patient was an elderly man with type 2 diabetes mellitus and chronic renal insufficiency, consistent with the typical high-risk profile for hypervirulent *K. pneumoniae* (hvKp) infection [9]. Diabetes is a recognized risk factor for *K. pneumoniae* liver abscess and invasive syndrome, and poor glycemic control may further predispose to multiple abscesses and secondary bloodstream infection [10]. The clinical course was characteristic of aggressive hvKp infection, with abrupt onset, markedly elevated inflammatory markers, rapid progression to severe biliary infection, and subsequent formation of a large intra-abdominal abscess, reflecting its marked invasiveness and suppurative potential.

Another notable feature was the development of postoperative stress ulcer bleeding and coagulation dysfunction, likely related to sepsis-associated coagulopathy, chronic renal failure–related platelet dysfunction, and increased bleeding risk from extensive surgical trauma and severe inflammation. This case highlights the importance of close perioperative coagulation monitoring and timely blood product support in severe hvKp infection.
